# Body Mass Index Above 35 Has Increased Risk of Complications but Still Achieves Clinically Meaningful Improvement in Patient-Reported Outcomes After Anterior-Based Total Hip Arthroplasty

**DOI:** 10.1016/j.artd.2025.101665

**Published:** 2025-03-15

**Authors:** Alexander S. Dash, Michael A. Hewitt, Richard A. Ruberto, Tiffany A. Smith, Carl L. Herndon, Nana O. Sarpong

**Affiliations:** Department of Orthopedic Surgery, Columbia University Irving Medical Center, New York, NY, USA

**Keywords:** THA, Anterior approach, Obesity, Complications, Patient-reported outcomes

## Abstract

**Background:**

Increased perioperative complications in obese patients undergoing total hip arthroplasty (THA) have previously been reported. There is a relative paucity of data evaluating these complications strictly in the context of anterior-based THA. In this study, we compare the outcomes following anterior-based THA as a function of body mass index (BMI).

**Methods:**

A 1:1 matched retrospective cohort study was conducted. Patients undergoing anterior-based THA from January 2022 to June 2024 with a BMI >35 kg/m^2^ were matched 1:1 based on age and sex to patients with a BMI <35 kg/m^2^ from our division registry. Demographic data, surgical details, complications (intraoperative and postoperative), and patient-reported outcome measures (PROMs: 12-Item Short Form P/M, Western Ontario and McMaster Universities Osteoarthritis Index-P/S/F) were collected and analyzed.

**Results:**

There were 280 patients included (140 per group). There were 27 postoperative complications in the BMI >35 kg/m^2^ group and 10 in the BMI <35 kg/m^2^ group (*P* < .01). There were 10 major complications (4 dislocations, 2 periprosthetic fractures, and 4 deep infections requiring incision and drainage) in the BMI over 35 kg/m^2^ group, with no major complications occurring in the lower BMI group. There were 3 intraoperative complications (periprosthetic fracture), all in patients with BMI >35 kg/m^2^. There was significant improvement in Western Ontario and McMaster Universities Osteoarthritis Index P/S/F scores and 12-Item Short Form pain in both groups at 3 months postoperatively, with greater improvements seen in the BMI >35 kg/m^2^ group.

**Conclusions:**

The present study found that despite significant differences in postoperative complications, there were significant improvements in PROMs in patients with a BMI above and below 35 kg/m^2^ who underwent anterior THA.

## Introduction

Primary total hip arthroplasty (THA) is the gold standard treatment to restore function and mobility for patients with end-stage hip osteoarthritis [1]. In 2019, THA constituted approximately 35% of all total joint arthroplasty, and the number of THAs in the United States is projected to be 700,000 per year by 2040 [2]. In an effort to maximize benefits for patients and improve outcomes, the ideal surgical approach for THA is a topic of ongoing study [3].

The posterolateral approach was previously the most common approach for THA. [4] In more recent years, anterior-based approaches for THA have increased in popularity, with early evidence suggesting that the direct anterior approach (DAA) may improve short-term outcomes due to decreased muscle disruption [5]. The DAA has also been associated with reduced hospital length of stay [5], which may help decrease the cost burden of the procedure [6]. Another anterior-based approach is the modified Watson-Jones, often called the anterior-based muscle-sparing (ABMS) approach, which has also been adopted with comparable outcomes [2,7,8].

The challenge with anterior-based approaches that is often described is the relatively reduced exposure of the femur and risk of periprosthetic femoral fractures [[9], [10], [11]]. This risk may be amplified when arthroplasty surgeons utilize the DAA for THA in patients with higher body mass index (BMI). Increased BMI has been identified as an important risk factor for complications following THA [12]. In the context of DAA, increased BMI may also increase the risk of infection. Prior retrospective studies assessing DAA-THA in obese patients reported an increased risk of reoperation and complications, though the overall rates are comparable to non-DAA approaches for patients with class II obesity or heavier (BMI >35 kg/m^2^) [13]. Other investigations have corroborated the impact of obesity on outcomes for patients undergoing DAA-THA [14,15], with class III obese patients having higher rates of deep infection (2.1%) compared to all other BMI categories (<1%) [14]. Due to the infrequent nature of most complications, however, the overall data are limited.

As such, the present study compares complication rates and postoperative patient-reported outcome measures (PROMs) in a cohort of class II or greater obese patients, defined as BMI >35 kg/m^2^, against a matched cohort of patients with a BMI <35 kg/m^2^. The goal of this study was to further understand the impact of BMI on anterior-based THA.

## Material and methods

### Trial design

The study is a 1:1 matched, retrospective cohort study.

### Ethical approval

Institutional review board approval (IRB#AAAA5922) was obtained before initiation of this study, and informed consent was not obtained due to this study having exempt status.

### Study participants

All patients undergoing primary THA at our institution by six fellowship-trained arthroplasty surgeons from January 2022 to June 2024 were reviewed for inclusion. Patients with a BMI over 35 kg/m^2^ who underwent primary THA by anterior-based approaches, including either the DAA or the modified Watson-Jones ABMS approach, were identified and matched 1:1 based on age (±5 years) and sex to patients with a BMI of less than 35 kg/m^2^ during the same study period. Patients <18 years old, revision THA, conversion of prior hip surgery to THA, and patients undergoing simultaneous, bilateral THA were excluded. Staged, bilateral THAs were included.

### Data collection

Demographic data, surgical details, complications (intraoperative and postoperative), and PROMs were all collected from patient records in our electronic medical record. Demographic data were obtained from patient charts and included age, sex, race, ethnicity, and surgical information. The American Society for Anesthesiologists (ASA) class was taken from anesthesia records as a surrogate for overall comorbidity burden. The PROMs collected included the 12-Item Short Form Health Survey (SF-12) mental (M) and physical (P) and Western Ontario and McMaster Universities Osteoarthritis Index (WOMAC) pain (P), stiffness (S), and function (F). All questions for the WOMAC were asked from a 0 (best) to 4 (worst) scale with the pain subscale having 5 questions, function subscale having 17 questions, and stiffness subscale having 2 questions [16]. Both surveys were reverse scaled on a 0 to 100 scale, with 0 indicating the worst function and pain and 100 correlating to the best function and pain.

Complications were evaluated through chart review up to the end of the study period. Both intraoperative and postoperative complications were collected and included. Complications included intraoperative and postoperative periprosthetic fractures, postoperative dislocation, and wound complications including dehiscence, delayed wound healing, and superficial and deep infections. All patients were evaluated for any subsequent reoperations including incision and drainage (I&D) and revision surgery. Complications were further broken down into major (revision surgery, periprosthetic fractures, dislocation, deep infection, and infection requiring I&D) and minor (superficial dehiscence, superficial infection, seromas, and lateral femoral cutaneous nerve palsy) categories.

### Statistical analyses

Statistical analyses were conducted using SAS (version 9.4, SAS Institute, Cary, NC). Matching of the study cohorts was performed with each case receiving a matching control of the same sex and age (±5 years). Descriptive statistics were performed for demographic variables. Distributions were evaluated for normality via the Shapiro-Wilk test and non-normally distributed variables were log-transformed as appropriate. For comparison of the two BMI cohorts, all categorical variables were analyzed via chi-squared analyses. For comparison between BMI categories, logistic regressions were used with the Tukey-Kramer adjustment for multiple comparisons. Differences in PROMs were taken from baseline to 3 months, and those continuous variables were analyzed via unpaired t-tests. Paired t-tests looked at differences in PROMs in each individual group over time. There was substantial loss to follow-up for the PROMs at 1 year; therefore, values for only baseline and 3 months were analyzed. Alpha value of 0.05 was used for significance. A *post-hoc* power analysis performed determined that there would need to be 120 patients per group to see a significant difference based on the major and minor complication rates found in this study (alpha of 0.05 and 80% power).

## Results

### Participant characteristics

There were 280 patients (140 cases and 140 controls) included in this study. Demographic variables are summarized in Table 1. In the overall cohort, 172 patients (61.0%) were females and 168 (59.0%) identified as non-Hispanic White. Mean values for age (62 ± 11) were identical between the groups (*P* = 1.00). As expected, BMI was different between the groups with the over 35 BMI group having a mean BMI of 38.5 ± 3.2 (range 35.0 to 49.8 kg/m^2^) and the under 35 BMI group having a mean of 26.8 ± 3.8 (range 15.0 to 34.8 kg/m2; *P* < .0001). The most common BMI category in the BMI<35 group was overweight with 67 patients with 31 patients being in the class I obesity category and 38 patients having healthy weight. In the BMI >35 group, 101 patients had class II obesity and the remaining 39 had class III obesity. ASA class was significantly worse in the BMI over 35 group (*P* < .0001).

**Table 1:** Demographic characteristics by BMI group and associated *P*-values.

Demographic characteristics	BMI > 35 kg/m^2^	BMI < 35 kg/m^2^	*P*-values
Age (mean ± SD)	62 ± 11	62 ± 11	1.000
BMI (mean ± SD)	39 ± 3	27 ± 4	<.0001
BMI categories (N, %)			<.0001
Underweight	4, 1%	0, 0%	
Healthy weight	38, 14%	0, 0%	
Overweight	67, 24%	0, 0%	
Class I obesity	31, 11%	0, 0%	
Class II obesity	0, 0%	101, 36%	
Class III obesity	0, 0%	39, 14%	
Sex (n, %)			1.000
Female	86, 61%	86, 61%	
Male	54, 39%	54, 39%	
Race (n, %)			.06
Asian	0, 0%	5, 4%	
Black or African American	24, 17%	21, 15%	
White	98, 70%	82, 58%	
Other	11, 8%	20, 14%	
Declined	7, 5%	12, 9%	
Ethnicity (n, %)			.40
Hispanic	15, 10%	22, 15%	
Non-Hispanic	115, 82%	105, 75%	
Unknown	10, 8%	12, 9%	
Declined	0, 0%	1, 1%	
ASA class (n, %)			<.0001
1	2, 1%	4, 3%	
2	46, 33%	90, 64%	
3	92, 66%	45, 32%	
4	0, 0%	1, 1%	

### Surgical details

Surgical characteristics were similar between groups and are summarized in Table 2. Most patients had cementless fixation (95.0%), with no difference between the groups (*P* = .39). Most patients (82.0%) underwent spinal anesthesia, which did not differ between groups (*P* = .56). There were 14 (5%) dual mobility bearings. Implant head sizes ranged from 22 to 40, with a majority (95%) being 32, 36, or 40 mm. When removing patients with 22- or 28-mm heads (dual mobility implants), there was no difference in head size between groups (*P* = .29). Nearly all (98%) patients received preoperative cefazolin, with 5 patients receiving vancomycin and/or clindamycin due to known penicillin allergy. Of these 5 patients, 4 were in the BMI under 35 kg/m^2^ and 1 was in the BMI over 35 kg/m^2^ group. Operative time was significantly longer in the BMI over 35 kg/m^2^ group (*P* < .05). Surgical approach did not differ between groups, with most patients (75.0%) undergoing THA through the DAA, of which 4, split 2 and 2 between the groups, were through a bikini incision.

**Table 2:** Surgical characteristics and complications and associated *P*-values.

Surgical characteristics	BMI > 35 kg/m^2^	BMI < 35 kg/m^2^	*P*-values
Laterality (n, %)			.63
Right	74, 53%	78, 56%	
Left	66, 47%	62, 44%	
Diagnosis (n, %)			
Osteoarthritis	124, 88%	119, 85%	.57
Avascular necrosis	7, 5%	13, 10%	.16
Fracture	5, 4%	6, 4%	.76
Other	4, 3%	2, 1%	.71
Cement usage (n, %)			.39
Cementless	135, 96%	132, 94%	
Cemented	5, 4%	8, 6%	
Femoral head size			.29
32	37, 28%	47, 35%	
36	89, 68%	86, 64%	
40	5, 4%	2, 1%	
Anesthesia type (n, %)			.56
General	30, 21%	21, 15%	
Spinal	110, 79%	119, 85%	
Surgical time (mean ± SD; min)	91 ± 32	79 ± 24	.0005
Operative room time (mean ± SD; min)	147 ± 39	129 ± 31	<.0001
Surgical approach (n, %)			.35
Direct anterior	99, 71%	112, 80%	
Modified Watson-Jones ABMS	41, 29%	28, 20%	
Major surgical complications (n, %)	10, 7%	0, 0%	.003
Minor surgical complications (n, %)	17, 12%	10, 7%	.16
Surgical complications (n, %)			NA
Dislocation	4, 3%	0, 0%	
Periprosthetic fracture	2, 1%	0, 0%	
Infection requiring I&D	4, 3%	0, 0%	
Superficial wound complication	12, 8%	5, 4%	
Wound dehiscence	1, 0%	1, 0%	
Seroma	3, 2%	1, 0%	
Lateral femoral cutaneous nerve paresthesia	1, 0%	1, 0%	
Other	0, 0%	1, 0%	

### Surgical complications

**Figure 1:**
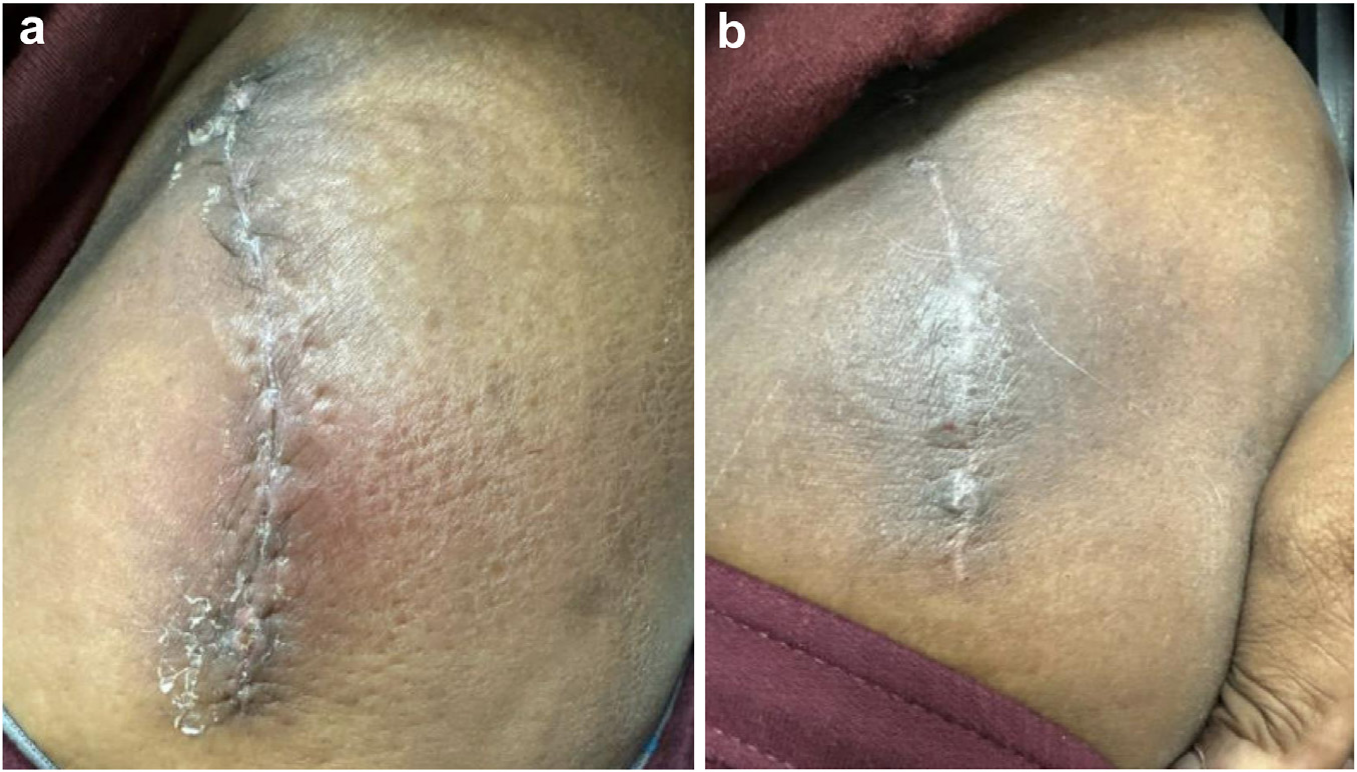
Minor superficial wound complication. Pictures and progression of minor superficial wound complication in patient with BMI over 35 kg/m^2^. (a) From the first follow-up visit ∼2 weeks postoperation with redness and minimal drainage. Aspiration at that time showed serosanguinous fluid, indicating a likely postoperative seroma without concern for infection. (b) Approximately 1 month later, showing resolution with successful wound healing and no drainage or signs of infection.

**Figure 2:**
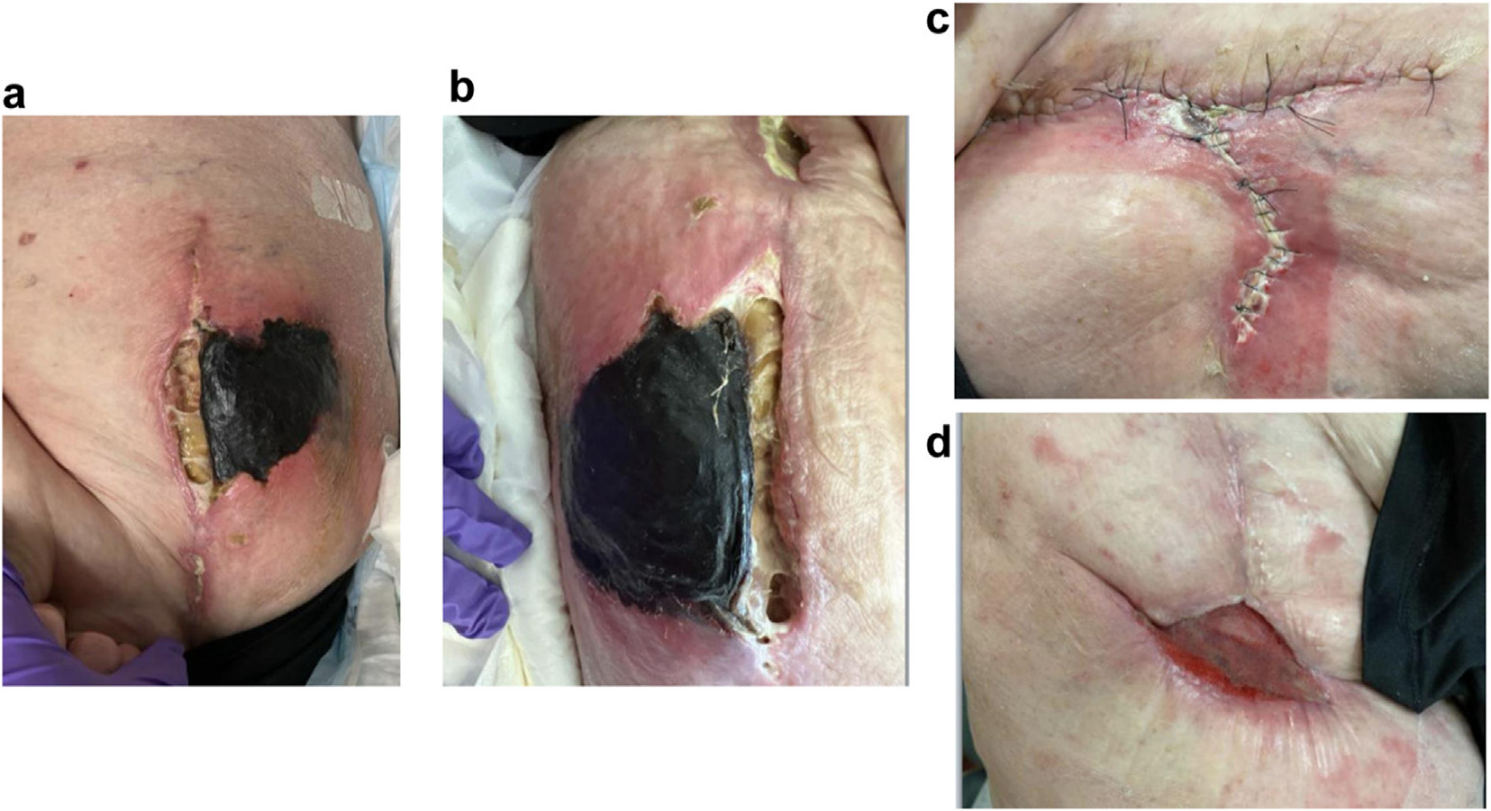
Major superficial wound complication. Pictures and progression of substantial requiring return to operating room for I&D. (a and b) Two pictures on the left show pictures of wound ∼1 month postoperation. Progression from (a and b) occurred over a few days, and substantial necrosis required surgical intervention. (c) In the sequence is 1 month status post first I&D with continued superficial infection requiring 2 more I&Ds. (d) The most recent clinical image with improved healing and clearance of infection. Hardware remained uninfected despite substantial superficial wound complications.

**Figure 3:**
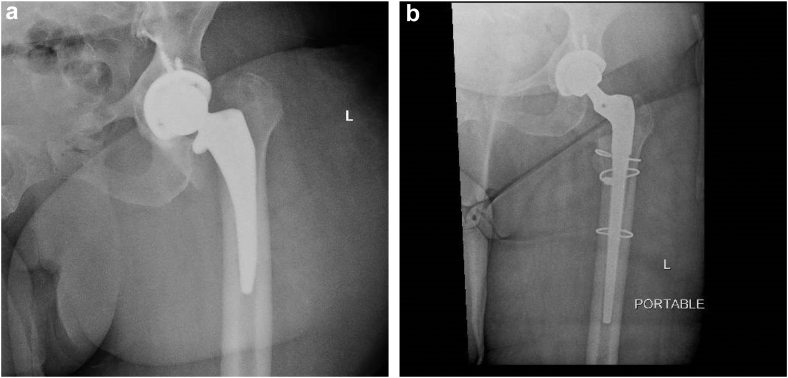
Radiographs of periprosthetic femur fracture and revision. Representative radiographs of a patient with periprosthetic femur fracture 1 month postoperation requiring revision. (a) An intertrochanteric periprosthetic fracture at the level of the implant with moderate displacement and comminution. (b) Postrevision radiographs with a new, long-stem revision prosthesis and 3 cerclage wires.

There were 27 (19.0%) patients in the higher BMI group and 10 (7.0%) patients in the lower BMI group with any complication (major or minor), with an odds ratio for any complication in BMI >35 group of 3.1 (95% confidence interval: 1.4-6.7, *P* < .01). While there was no difference in minor complications between the groups (*P* = .15), there was a significantly higher rate of major complications in the BMI >35 group (*P* < .01). There were no major complications in the BMI <35 and no subsequent reoperations or revision surgery. In the BMI <35 group, there was no difference in complications when comparing individual groups (underweight, healthy weight, overweight, and class I obesity; all *P-value* > .05). In the BMI >35 group, there were 4 (3.0%) dislocations and 2 (1.0%) femoral periprosthetic fractures, of which 4 required revision surgery. There were 4 (3.0%) patients in the BMI over 35 group who underwent I&D for superficial soft tissue infection. Overall and specific surgical complications per group are outlined in Table 2. Figure 1, Figure 2, Figure 3 show representative images of minor wound complication, major wound complication requiring I&D, and radiographs of a periprosthetic fracture before and after revision, respectively. No prosthetic joint infections requiring revision were reported in either group. The remainder of the complications were classified as minor, including 1 lateral femoral cutaneous nerve paresthesia and the remainder being wound-related complications with wound dehiscence, incision cellulitis, seroma development, or delayed wound healing. There were 3 (2.0%) intraoperative fractures reported, all occurring in patients in the BMI over 35 kg/m^2^ cohort.

### Patient-reported outcome measures (PROMs)

The change in PROM scores are summarized in Table 3. There were 126 (90.0%) patients in both groups who completed the preoperative SF-12 surveys with 122 in the BMI under 35 kg/m^2^ and 124 in the BMI over 35 kg/m^2^ group completing the WOMAC surveys preoperatively. There were 44 (31.0%) patients in each group who completed the SF-12 and WOMAC surveys at 3 months. At 1 year, less than 15% of each group completed these surveys. All preoperative PROMs were equivalent between the groups, apart from better preoperative SF-12 pain scores in the BMI under 35 group (40 vs 37; *P* < .05).

**Table 3:** Change in patient-reported outcome measures between baseline and 3 months.

PROM measure	BMI > 35 kg/m^2^ baseline to 3 months	BMI < 35 kg/m^2^ baseline to 3 months	*P*-values
SF-12 P (mean ± SD)	9.5 ± 7.4	5.5 ± 7.9	.02
SF-12 M (mean ± SD)	0.7 ± 9.4	0.8 ± 8.8	.93
WOMAC pain (mean ± SD)	43.5 ± 22.7	32.6 ± 23.2	.03
WOMAC function (mean ± SD)	34.4 ± 26.4	32.1 ± 26.5	.70
WOMAC stiffness (mean ± SD)	38.5 ± 21.0	27.7 ± 23.7	.03

**Figure 4:**
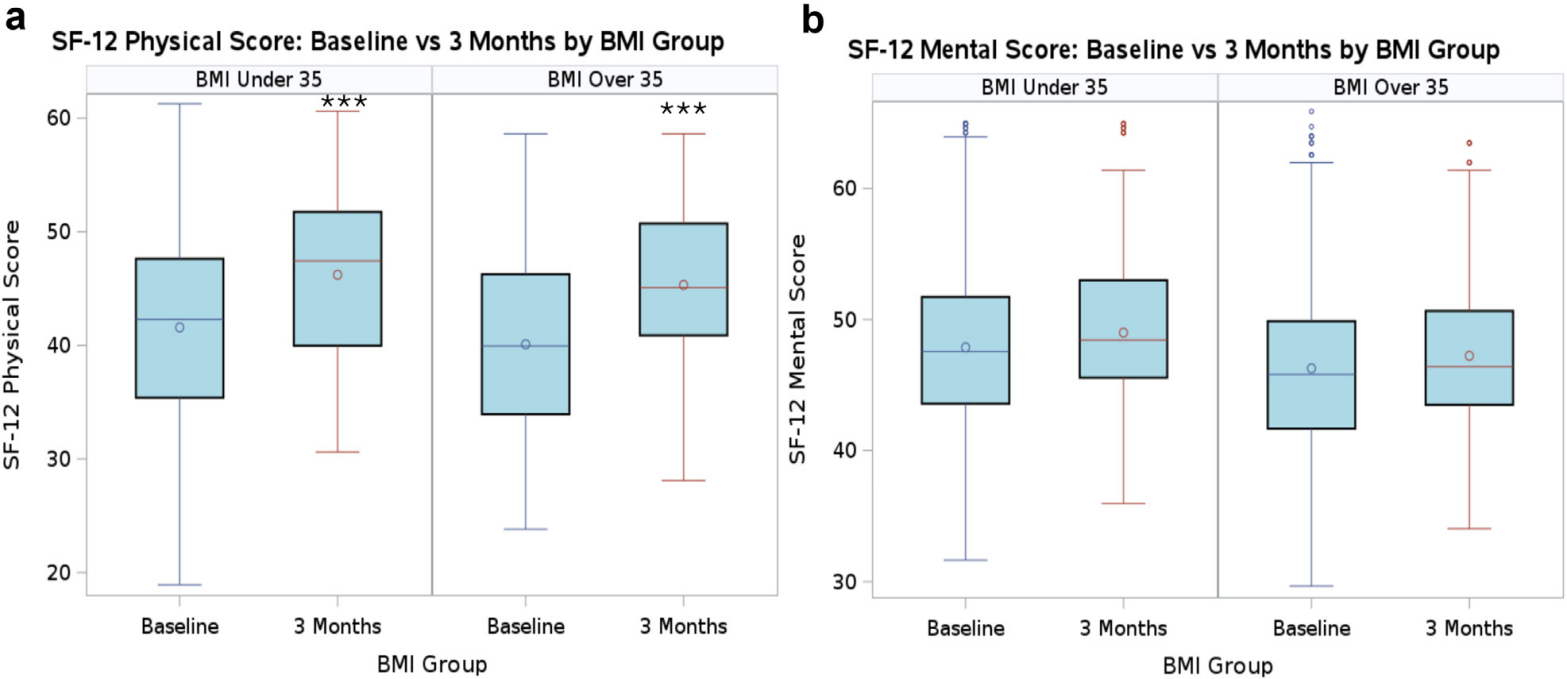
Box and whisker plot of SF-12 scores at baseline and 3 months. Box and whisker plot of (a) SF-12 Physical and (b) Mental scores in the BMI over 35 kg/m^2^ and under 35 kg/m^2^ groups at baseline and 3 months postoperatively. ∗∗∗ indicates *P*-value <.001.

**Figure 5:**
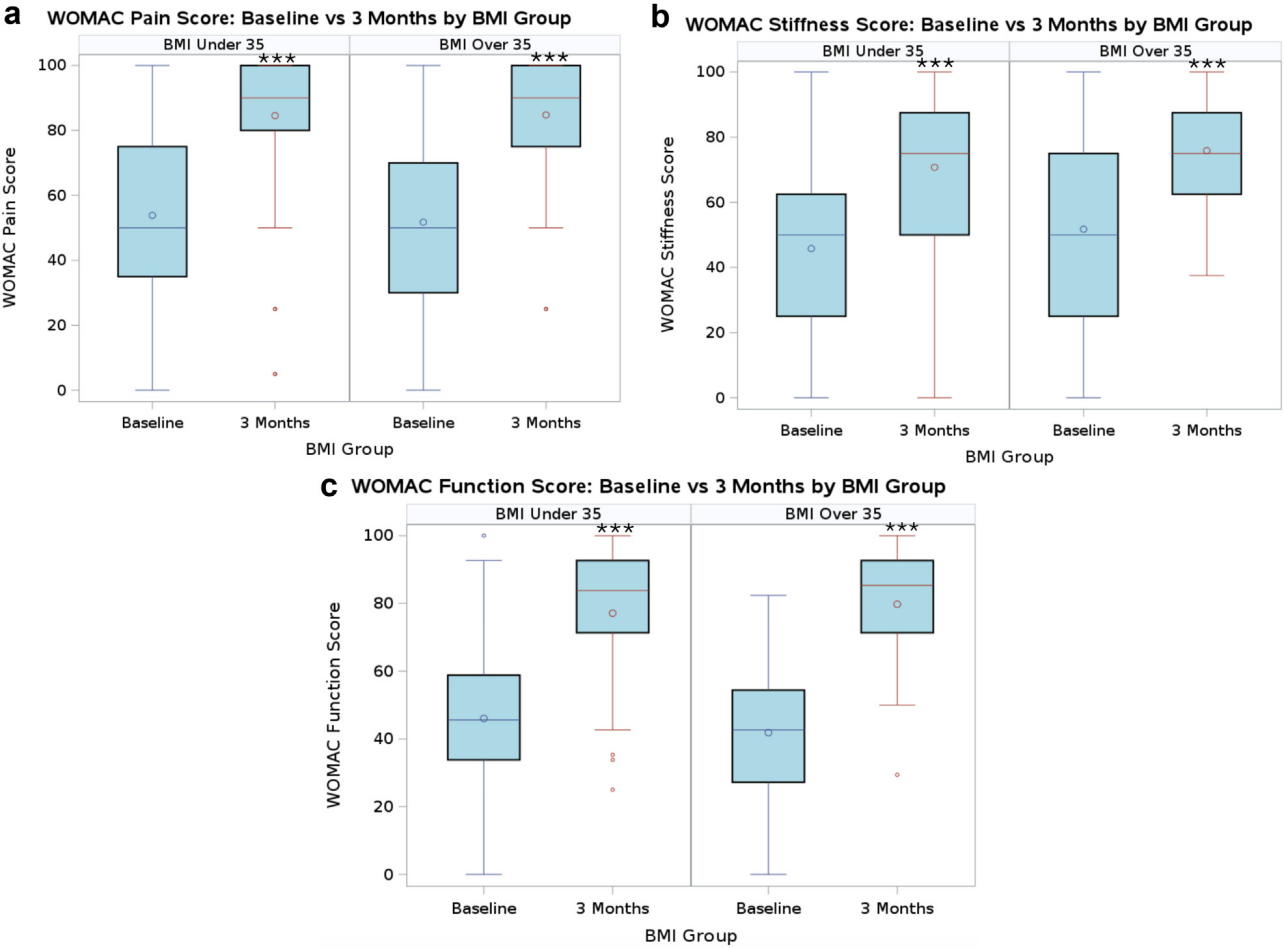
Box and whisker plot of WOMAC scores at baseline and 3 months. Box and whisker plot of WOMAC (a) pain, (b) function, and (c) stiffness scores in the BMI over 35 kg/m^2^ and under 35 kg/m^2^ groups at baseline and 3 months postoperatively. ∗∗∗ indicates *P*-value <.001.

Figure 4 shows the change in SF-12 P and SF-12 M scores from baseline to 3 months. Paired t-tests within each group revealed that SF-12 M scores did not change from baseline to 3 months in either group, but SF-12 P scores improved in both groups from baseline to 3 months postoperatively (*P* < .001). Unpaired t-tests revealed no difference in change in SF-12 M scores, but that the BMI over 35 group had a larger improvement in SF-12 P scores compared to the BMI under 35 group (Table 3). Figure 5 shows the change in WOMAC P, S, and F scores from baseline to 3 months in the BMI over and under 35 groups. The WOMAC P, F, and S scores improved in each group between baseline and 3 months (*P* < .001). The WOMAC S scores improved similarly between groups from baseline to 3 months; however, in the BMI over 35 group, there was a significantly larger improvement in WOMAC P (*P* = .03) and F (*P* = .03) scores compared to the BMI under 35 group (Table 3). At 3 months, SF-12 and WOMAC scores were similar between the groups.

## Discussion

This study found that patients with a BMI over 35 kg/m^2^ were significantly more likely to experience complications during anterior-based THA. While major complications and revision surgery were reported in less than 10% of patients overall, all occurred in the BMI over 35 group. Within the BMI under 35 group, there was no difference in complications between BMI categories, supporting our use of 35 as our cutoff. Additionally, intraoperative complications were seen strictly in the BMI over 35 group. Despite this, patient-reported outcomes, SF-12 pain and WOMAC pain, function, and stiffness in both groups were significantly improved from baseline to 3 months after surgery. There were greater or similar improvement in PROMs from baseline to 3 months between the BMI over 35 group compared to the BMI under 35 group, providing evidence that both BMI cohorts had substantial self-reported improvement after anterior-based THA.

Traditionally, anterior approaches to the hip were not as commonly performed in obese patients given the difficulty with femoral exposure and increased rates of infections and wound complications [14,15,[17], [18], [19]]. Wound complications were reportedly related to the pannus overlying the incisional site, thinner skin overlying the incisional site, and proximity of genitalia to the surgical site [9,18,20,21]. Multiple studies have reported higher infection risks and rates of wound complications in obese patients undergoing anterior THA compared to nonobese counterparts, with morbidly obese patients (BMI >40 kg/m2) experiencing the highest rates of complications [[13], [14], [15],^17^,^22^]. Even with these differential complication rates, PROMs are consistently and substantially improved in the obese patients after receiving anterior approach THA, much likely as seen in our study [13,15,22]. For the WOMAC subscales, the minimal clinically important difference, or smallest change in PROM scores to result in a clinically meaningful change for our patients, has been found to be between 10 and 20 [23,24]. Each WOMAC subscale in our study improved by over 30 points, which corroborates that there can be marked clinical improvement with the anterior approach for THA in this population. Furthermore, patients with a BMI over 35 kg/m^2^ had greater or equivocal improvements in their PROMs from baseline to 3 months in all our subscores. While our response rate was relatively low, this overwhelmingly positive result in PROMs shows that our patients with class II obesity or greater, on aggregate, have marked clinical improvement and therefore likely do not need to have access to this procedure restricted.

The findings reported in this study are especially important as the DAA and ABMS have become more popular over recent years. Over 50% of arthroplasty surgeons at the most recent Annual Meeting of the American Association of Hip and Knee Surgeons report using the DAA in their practice [25]. The perceived benefits of the anterior approach involve its use of an intermuscular plane, which allows for fewer releases of muscle tendons around the hip. While the posterior THA approach has traditionally required postoperative precautions, including limited hip adduction, internal rotation, and flexion to minimize the risk of dislocation [26], the anterior approach does not commonly require such precautions. While there is some evidence of decreased dislocation rates with the anterior approach, most of the literature has not found significant differences [[27], [28], [29], [30], [31]]. The anterior approach, however, has been shown to have decreased early postoperative pain, shorter hospital length of stay, and faster recovery after THA, although these differences do not persist, and outcomes are equivocal with longer follow-up times [[32], [33], [34]].

There may be some benefit to using the posterior approach in more obese patients if there are serious concerns about infection or a history of infection; however, this study validates findings from prior studies that self-reported outcomes do not differ based on BMI. Deep infection rates in 1 study were found to be equivocal between the anterior and posterior approaches for patients with BMI less than 35 kg/m^2^ (0.28% vs 0.36%) and for patients with a BMI over 35 kg/m^2^ (2.35% vs 2.70%) [35]. Our study, albeit smaller than that cohort, had no deep infections requiring revision. Another small prospective study showed an increased risk of overall complications (22% vs 5%) in morbidly obese (BMI >40 or BMI >35 with significant comorbidity) vs nonobese patients in the anterolateral approach [36]. Furthermore, there was no significant difference in reoperation rates between the groups, providing evidence that obesity, not approach, is likely the most important risk factor for these patients [35]. We observed a 3% reoperation rate for patients in our BMI over 35 cohort for superficial infections, with successful resolution, with no revisions for infection. The patient shown in Figure 2 did require I&D and had substantial skin necrosis. While this was an abnormal and relatively catastrophic soft tissue infection, it did not require revision and only required multiple superficial washouts. This is an example of a patient who may have benefited from a posterior approach; however, with her body habitus and comorbidities, it is hard to solely attribute this infection to her hip approach.

It is important to recognize and emphasize that several confounders may be present when investigating obesity and complication profiles after surgery [[37], [38], [39], [40]]. Part of this may be in part due to increased rates of comorbidities and thus higher ASA scores in obese patients [38,40,41]. In our study, despite ages being normalized, ASA scores were significantly higher in the BMI over 35 cohort, an indication that this group was deemed to be at greater risk of complications than their age- and sex-matched peers with lower BMI. Higher comorbidity burden is known to contribute to worse healing and infection rates [42] and higher BMIs, paradoxically, have been shown to have negative impacts on bone metabolism and structure that could lead to greater rates of mechanical complications such as periprosthetic fracture [43]. Therefore, some of the difference in outcomes can likely be attributed to the baseline difference in overall health risk that existed between the groups.

Modified approaches and incisions, particularly the “bikini” incision in the anterior approach, have been found to reduce the risk of wound complications and infection rates [15,21], possibly by decreasing the contact between the overlying pannus and the incisional site as well as being in line with Langer’s lines, which are theorized to decrease incisional tension and improve wound healing [21,44,45]. The bikini incision is a horizontal incision just under the flexor crease, with 1 of 3 being lateral and 2 of 3 being medial to the anterior superior iliac spine that allows surgeons to gain access to the same muscular planes as the traditional longitudinal incision of the DAA [45]. These adjustments and future considerations are important, as they may modify risk and decrease the rates of complications seen in our current study, as most of the patients in this cohort had the traditional DAA or modified Watson-Jones approach with a longitudinal incision.

There are important limitations to this study. First, this is a retrospective cohort study from a single institution that primarily performs THA through the anterior approach, making it subject to the inherent limitations of all retrospective reviews. There is no comparison to posterior approaches, so conclusions cannot be drawn on if these patients would have had different outcomes with the posterior approach. There were relatively low response rates to the PROMs, and future work will need to ensure more complete collection of postoperative PROMs. Finally, while all patients received an anterior hip approach, there were slight variations as some patients received a modified Watson-Jones while others received the DAA. Outcomes and complications have typically been shown to be equivalent between the two techniques [46]; it cannot be ruled out that nonstandard technique could impact the results of this study.

## Conclusions

Despite higher complication rates among patients with a BMI over 35 undergoing anterior THA, there were comparable PROMs 3 months postoperatively. With an overall relatively low major complication rate, the present study adds to the growing body of literature that anterior THA can be performed safely and effectively, even in class II and above obese patients, and that surgeon comfort should be the most important factor in guiding preoperative planning of the approach. It is critical, however, to discuss with patients who have a BMI over 35 that they are at an increased risk for complications and to discuss the risks and benefits of using an anterior over the posterior approach in these cases. Future studies should focus on evaluating ways to mitigate the risk of complications in obese patients who require THA and on developing strategies to identify those at greatest risk for complications.

## Conflicts of interest

N. O. Sarpong is a paid consultant for Link Orthopaedics. All other authors declare no potential conflicts of interest.

For full disclosure statements refer to https://doi.org/10.1016/j.artd.2025.101665.

## CRediT authorship contribution statement

**Alexander S. Dash:** Writing – review & editing, Writing – original draft, Methodology, Investigation, Formal analysis, Data curation, Conceptualization. **Michael A. Hewitt:** Writing – review & editing, Writing – original draft, Investigation, Formal analysis, Data curation. **Richard A. Ruberto:** Writing – review & editing, Methodology, Investigation, Data curation, Conceptualization. **Tiffany A. Smith:** Writing – review & editing, Methodology, Investigation, Data curation, Conceptualization. **Carl L. Herndon:** Writing – review & editing, Visualization, Supervision, Resources, Data curation, Conceptualization. **Nana O. Sarpong:** Writing – review & editing, Writing – original draft, Validation, Supervision, Resources, Methodology, Investigation, Data curation, Conceptualization.

## Appendix A Supplementary data

Conflict of Interest Statement for Dash

Conflict of Interest Statement for Herndon

Conflict of Interest Statement for Hewitt

Conflict of Interest Statement for Sarpong

Conflict of Interest Statement for Ruberto

Conflict of Interest Statement for Smith
